# Advance in the application of organoids in bone diseases

**DOI:** 10.3389/fcell.2024.1459891

**Published:** 2024-09-03

**Authors:** Yajie Kong, Yujia Yang, Yu Hou, Yuzhong Wang, Wenjing Li, Yongzhou Song

**Affiliations:** ^1^ Department of Orthopedics, The Second Hospital of Hebei Medical University, Shijiazhuang, Hebei, China; ^2^ Hebei Medical University-National University of Ireland Galway Stem Cell Research Center, Hebei Medical University, Shijiazhuang, Hebei, China; ^3^ Hebei Research Center for Stem Cell Medical Translational Engineering, Hebei Medical University, Shijiazhuang, Hebei, China; ^4^ Department of Oral Medicine, The Second Hospital of Hebei Medical University, Shijiazhuang, Hebei, China; ^5^ Hebei Key Laboratory of Rare Disease, Shijiazhuang, Hebei, China

**Keywords:** bone diseases, bone organoids, stem cells, regenerative medicine, biomaterials

## Abstract

Bone diseases such as osteoporosis and osteoarthritis have become important human health problems, requiring a deeper understanding of the pathogenesis of related diseases and the development of more effective treatments. Bone organoids are three-dimensional tissue masses that are useful for drug screening, regenerative medicine, and disease modeling because they may mimic the structure and physiological activities of organs. Here, we describe various potential methods for culturing bone-related organoids from different stem cells, detailing the construction processes and highlighting the main applications of these bone organoid models. The application of bone organoids in different skeletal diseases is highlighted, and current and promising bone organoids for drug screening and regenerative medicine as well as the latest technological advancements in bone organoids are discussed, while the future development of bone organoids is discussed. Looking forward, it will provide a reference for constructing bone organoids with more complete structures and functions and applying them to biomedical research.

## Graphical Abstract

**Figure:**
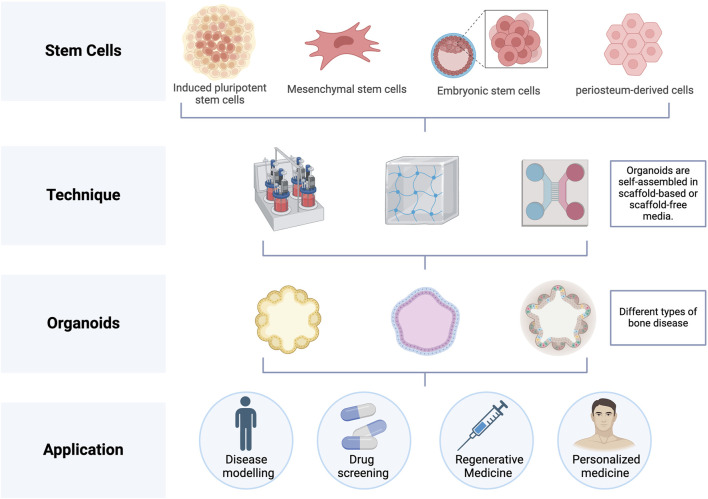
The graphical abstract was created with BioRender (www.biorender.com).

## 1 Introduction

Bone diseases have been affecting tens of millions of individuals around the world, especially diseases with long recovery periods such as osteoporosis, osteoarthritis (OA) and bone-related cancers, which have brought a huge economic burden (Xu et al., 2018). As the elderly population grows, the incidence of these diseases increases year by year, which has a serious impact on patients’ quality of life and physical and mental health (Bouvard et al., 2021; Hunter et al., 2020). On the other hand, the pathophysiology of bone disorders is still not fully understood. Preclinical bone research currently relies heavily on animal models and two-dimensional mammalian cell cultures. However, traditional two-dimensional (2D) cell culture models have many limitations, such as the lack of interactions between cells and cells and matrix *in vivo* (Duval et al., 2017), and the inability to accurately simulate the physiological microenvironment *in vivo* (Lin et al., 2019). Although animal models more closely mimic the natural biological environment, their establishment is complex, time-consuming, and labor-intensive. Additionally, physiological differences between animals and humans pose challenges to clinical translation (Samvelyan et al., 2020). In addition, bone defects are also a clinical problem. Bone defects caused by trauma, infection, tumors, etc. require bone transplantation or artificial bone materials to repair (Dimitriou et al., 2011). However, these methods face challenges such as limited availability of sources, risk of infection, immune rejection, and inadequate healing. Therefore, to overcome the limitations of traditional research methods, people began to explore more advanced research technologies, such as *in vitro* three-dimensional cell culture systems. This *in vitro* three-dimensional (3D) system can more accurately simulate the interaction between cells and matrix in the body, provide a research platform that is closer to the real biological *in vivo* environment, and is expected to become an effective alternative to bridge the gap between traditional two-dimensional cell culture and animal models (Kamm et al., 2018).

Bone organoids are 3D self-renewing and self-organizing micro-bone tissues grown *in vitro* from progenitor cells (such as osteoblast progenitors) or stem cells (including bone stem cells, embryonic stem cells, etc.), and can also incorporate differentiated cells like osteoclasts to mimic bone remodeling (Fatehullah et al., 2016). They can simulate the complexity of bone tissue and its remodeling process in a high-fidelity and controllable way and are designed to simulate the effects of microgravity and degeneration on trabecular bone (Park et al., 2021; Iordachescu et al., 2021). Various biocompatible materials, including Matrigel and synthetic alternative hydrogels, are utilized in the production of bone organoids to support their self-organization. Unlike traditional bone tissue engineering, the construction of bone organoids relies on the differentiation and self-organizing capabilities of stem cells. It uses a series of methods such as bioactive materials and bioreactors to guide cells to gradually differentiate and self-organize under the influence of microenvironmental signals, assembling into bone microtissues *in vitro* rather than inducing stem cell differentiation and achieving bone regeneration through a combination of seed cells, scaffold materials, and growth factors (Yin et al., 2016). By mimicking the *in vivo* development of bone tissue, bone organoids not only offer novel strategies for bone defect repair but also more accurately replicate the structure and function of bone. This provides a valuable research platform for studying bone diseases, testing drugs, and advancing basic orthopedic research. Latest research shows that researchers have successfully developed self-assembled human skeletal organoids that can be used for disease modeling and drug testing and display spontaneous polarization of cartilage and bone components (Abraham et al., 2021). From a materials biology perspective, hydrogels have been studied as materials for building bone organ tissue (Wu et al., 2023). Additionally, dental pulp stem cells have been investigated as a potential vascular source that could improve the survival of bone marrow stem cells within bone organoids (Li et al., 2023). The complexity of bone organoids and the heterogeneity of osteogenic stem/progenitor cells pose challenges for constructing organisms that model the skeletal system (Qing et al., 2023). To guide future research in this field, current efforts focus on evaluating skeletal stem cells, environmental variables, and potential organoid culture media candidates (Qing et al., 2023). Bone has inherent regenerative ability, and bone organoids provide a platform for studying bone regeneration technology (Zhao et al., 2023). The construction and application of bone organoids show broad prospects in organ development, drug screening and mechanism research (Chen et al., 2022a). This review focuses on recent advances in bone organoids, focusing on their applications in different skeletal diseases, and then describes the importance of bone organoids in the field of regenerative medicine. Finally, some perspectives and perspectives in bone organoid development are discussed.

## 2 Generation of bone organoids

**FIGURE 1:**
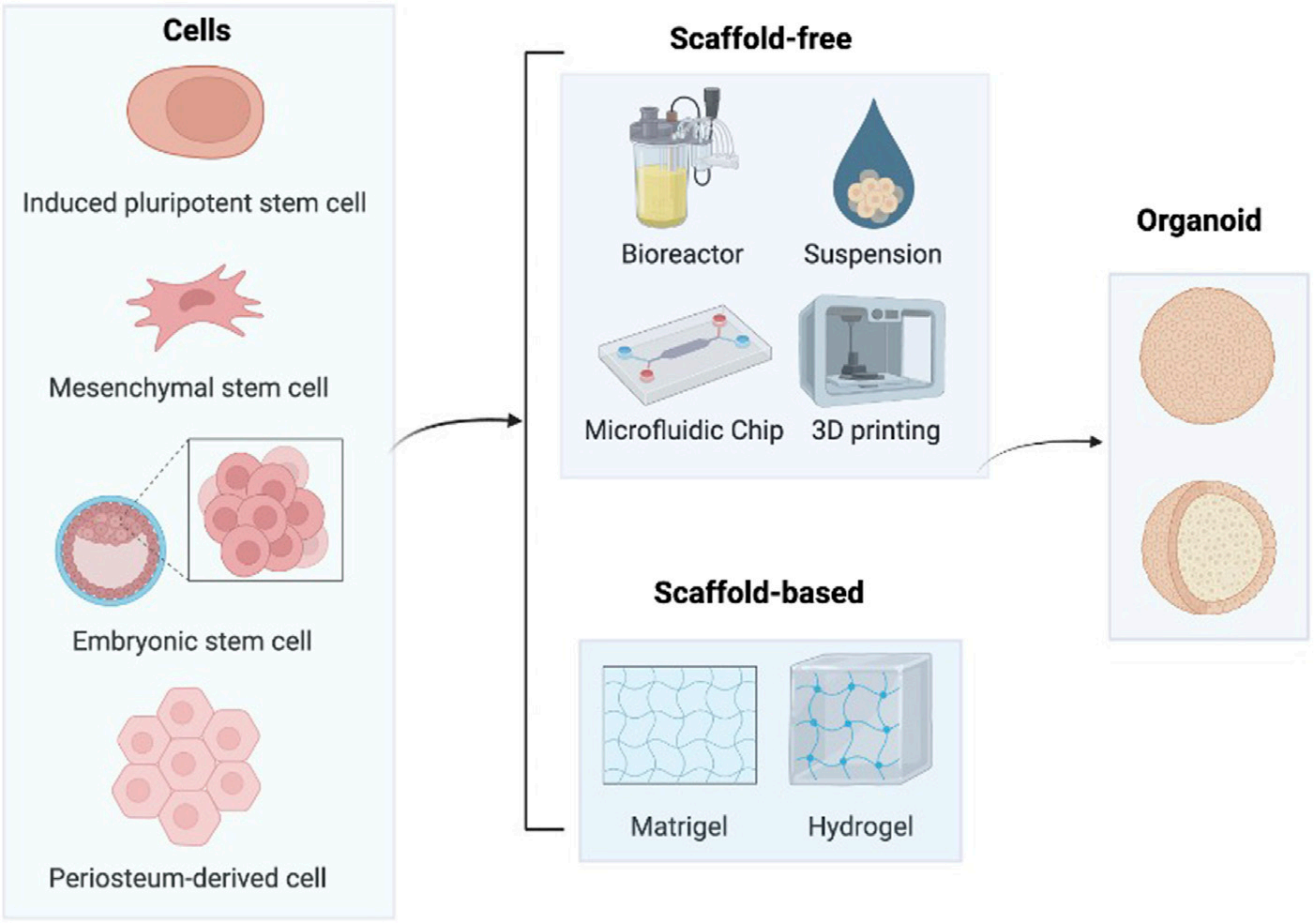
Construction process of bone organoids. In order to cultivate cartilage organoids, stem cells such as iPSCs, MSCs, ESCs, and hPDCs are primarily utilized. After that, these cells self-assemble to create organoids in medium with or without scaffolds. Image created with BioRender (www.biorender.com).

Bone organoids are 3D, self-renewing, and self-organizing miniaturized bone tissues derived from stem cells or progenitor cells, exhibiting biomimetic spatial properties. They are developed using bioactive materials (Fatehullah et al., 2016). A range of biocompatible materials, including hydrogels, is used to support the self-organization of bone tissue, facilitating the creation of biomimetic structures. Establishing bone organoids involves several key steps (Figure 1). First, the appropriate cell source must be selected. Next, biomaterials are introduced as a supportive matrix to facilitate the growth and differentiation of the cells into 3D organoids. Finally, specific construction techniques are applied to shape the organoids into 3D spherical structures.

### 2.1 Stem cell model

Stem cell models are essential tools in biomedical research and regenerative medicine, providing critical insights into human development and disease mechanisms. Organoids, three-dimensional structures derived from various stem cell types, serve as valuable models for these studies. They can be generated from human induced pluripotent stem cells (hiPSCs), which are adult cells reprogrammed to a pluripotent state capable of differentiating into any cell type; human mesenchymal stem cells (hMSCs), which can differentiate into bone, cartilage, and fat cells; human embryonic stem cells (hESCs), pluripotent cells derived from early-stage embryos; and human periosteum-derived cells (hPDCs), which are obtained from the periosteum and are notable for their potential in bone and cartilage regeneration. These diverse stem cell origins enable the modeling of complex tissues and the exploration of cellular behaviors in a controlled laboratory setting.

### 2.2 Induced pluripotent stem cell

iPSCs are populations reprogrammed from fibroblasts with the potential to differentiate into a wide variety of cell types. In 2018, new discoveries were made in bone tissue engineering, and iPSC-derived organ tissues have great potential to treat fractures and bone defects (Perez et al., 2018). Researchers have utilized iPSCs as building blocks for bone implants aiming to promote bone repair by inducing osteoblast differentiation (Perez et al., 2018), emphasizing the importance of iPSC-based approaches in addressing delayed fracture healing and segmental bone defects. Connor et al. (2020) created osteochondral organoids using mouse induced pluripotent stem cells to study cartilage-bone interactions, providing insights for modeling joint diseases such as osteoarthritis (Connor et al., 2020). The development of iPSCs-derived hematopoietic organoids and hiPSCs-derived bone organoids in 2022 has shown promise in creating complex bone organoids for disease modeling and discovery (Frenz-Wiessner et al., 2023; Frenz et al., 2022). In 2022, The ability of iPSCs as cartilage organoid starting cells was validated by the researchers’ effective induction and characterization of iPSCs to develop into cartilage organoids *in vitro* (Zhong et al., 2022). Additionally, in 2024, researchers discovered that one way to generate bone marrow-like organoids (BMOs) is to use iPSCs to create a complex bone marrow niche to sustain lifelong hematopoiesis (Frenz-Wiessner et al., 2024). This approach has the potential to be a breakthrough in generating sufficient numbers of osteoblasts for a deeper understanding of bone and joint diseases (Huang et al., 2023). IPSCs are now a potent cell source for creating cartilage organoids as a result.

### 2.3 Mesenchymal stem cell

hMSCs were initially extensively studied in bone tissue engineering and have since been found to express and secrete a range of bioactive factors that play anti-inflammatory and immunoregulatory roles (Fu et al., 2017). Recent research has focused on integrating MSCs into organoid systems for applications in various fields. For example, the use of bone morphogenetic protein-2 loaded scaffolds to construct bone organoids *in vivo* has been shown to encourage the development of therapeutically beneficial cells, including MSCs (Dai et al., 2023). In 2023, some researchers have provided a protocol for isolating functional MSCs from bone organisms *in vivo*, providing an alternative to autologous MSCs (Deng et al., 2023). Co-culture of bone marrow MSCs with hematopoietic cells has been shown to support the growth of hematopoietic cells in bone marrow organ tissues, although current methods still have generation limitations (Khan et al., 2023). The latest research in 2024 shows that basement membrane extract enhances the endochondral ossification phenotype of MSCs cartilage tissue (Hinako et al., 2024). Taken together, these findings indicate that MSCs-derived bone organ tissue has great potential for regenerative medicine, disease modeling, and therapeutic applications.

### 2.4 Embryonic stem cell

ESCs are pluripotent cells capable of indefinite proliferation and differentiation into various lineages *in vitro*. They are derived from primitive gonads or early mammalian embryos. The study of Xu et al. (2002) confirmed that bone morphogenetic protein 4 (BMP4) can induce the differentiation of human ES cells into trophoblast cells, indicating that the use of specific growth factors can drive stem cells to differentiate into desired cell lines (such as bone cells). Research by Karp et al. (2006) pointed out that when hESCs are cultured *in vitro*, osteogenesis can be promoted if the embryoid body stage is removed. This finding suggests that by regulating the culture conditions of hESCs, they can be directed to differentiate into specific cell types, such as osteoblasts, which are critical for the development of hESC-derived bone organ tissue. In addition, Demirci et al. (2020) discussed the generation of hematopoietic stem/progenitor cells from human embryonic stem cells and beyond through organoid-induced differentiation. Lauren et al. (2021) successfully constructed craniofacial cartilage organoids by differentiating ESCs became neural crest stem cells, then proceeded with more differentiation and self-organization (Foltz et al., 2024). The strong expression of cartilage marker proteins in cartilage organoids, as revealed by mass spectrometry analysis, provides new insights into the development of craniofacial cartilage and cell signaling. This indicates that by adjusting the culture conditions of human embryonic stem cells, their potential to differentiate into specific cell lineages, such as bone cells, can be enhanced.

### 2.5 Human periosteum-derived cell

Bone tissues and organs derived from hPDCs have garnered significant attention in regenerative medicine and tissue engineering. Periosteum-derived cells, especially hPDCs, have been shown to have the potential for osteogenic differentiation and bone formation (Park et al., 2021a). These cells are an important source of tissue regeneration that can be extracted from people having surgery for issues relating to their bones (Bolander et al., 2020). In 2021, the development of organoid-based cartilage implants using hPDCs has also achieved promising results in tissue regeneration (Gabriella et al., 2021). These implants exhibit region-specific characteristics like native cartilage. Furthermore, the use of acellular cartilage ECM culture coating in 2022 has been shown to drive rapid and efficient bone organogenesis using hPDCs (Vas et al., 2022). The latest research in 2023 have shown that hPDCs have osteogenic differentiation capacity when exposed to factors such as BMP-9 (Park et al., 2023). This highlights the potential of utilizing hPDCs in bone tissue engineering applications. These studies highlight the great potential of utilizing hPDCs to create functional tissue constructs.

We compared various bone tissue organoid protocols in Table 1.

**TABLE 1:** Detailed comparison of bone tissue organoid protocols.

Protocol	Cell Source	Scaffold/Matrix	Culture	Key Features	Applications	Limitations
Protocol A	Human Mesenchymal Stem Cells (hMSCs)	Matrigel	3D bioprinting, growth factors (e.g., BMP-2)	High reproducibility, rapid osteogenesis	Bone regeneration, fracture healing	Limited vascularization, scalability issues
Protocol B	Human Induced Pluripotent Stem Cells (hiPSCs)	GelMA (Gelatin Methacrylate)	Microfluidic devices, perfusion culture	Mimics bone microenvironment, dynamic nutrient flow	Drug screening, toxicity testing	High cost, complex fabrication
Protocol C	Human Embryonic Stem Cells (hESCs)	PLGA [Poly(lactic-co-glycolic acid)] scaffold	Static culture, osteoinductive medium	Strong osteogenic potential, scalable production	Disease modeling, bone tissue engineering	Scaffold degradation, immune response potential
Protocol D	Human Periosteum-Derived Cells (hPDCs)	Fibrin gel	Dynamic culture, osteoinductive supplements (e.g., dexamethasone, ascorbic acid)	High osteogenic potential, strong cellular differentiation	Bone regeneration, periosteal tissue engineering	Limited scalability, variable cell yield
Protocol E	Bone Marrow-Derived Stem Cells (BMSCs)	Decellularized bone matrix	Static and dynamic culture systems	Natural bone matrix, supports vascularization	Regenerative medicine, transplantation studies	Limited donor availability, complex preparation
Protocol F	Primary Osteoblasts	Collagen hydrogel	Rotary cell culture, osteogenic differentiation factors	Enhanced mineralization, physiological relevance	*In vitro* bone formation studies, biomaterial testing	Limited lifespan of primary cells, donor variability

Notes: hMSCs, Human mesenchymal stem cells; hiPSCs, Human induced pluripotent stem cells; GelMA, Gelatin methacrylate; hESCs, Human embryonic stem cells; PLGA, Poly(lactic-co-glycolic acid); hPDCs, Human periosteum-derived cells; BMSCs, Bone marrow-derived stem cells.

This table provides a clear overview of the current methodologies in bone organoid research and their respective strengths and weaknesses, offering valuable insights for future developments in the field. To address the future perspectives in the field of bone tissue organoids, it is crucial to focus on several key areas. First, advancing the scalability and reproducibility of organoid protocols will be essential for translating these models from research to clinical applications. This involves refining the techniques for generating organoids from diverse cell sources and optimizing scaffold materials to better mimic the complex microenvironment of human bone tissue. Additionally, enhancing the integration of bone organoids with vascular and immune components will improve their physiological relevance and functionality. Future research should also explore personalized approaches, utilizing patient-specific cells to develop tailored treatments for bone diseases. Moreover, the development of high-throughput screening methods and advanced imaging technologies will facilitate the assessment of drug efficacy and toxicity in bone organoids. By addressing these challenges, bone tissue organoids have the potential to revolutionize bone regeneration and repair, offering innovative solutions for treating bone diseases and injuries more effectively.

## 3 Tissue explant culture and biomaterials

**FIGURE 2:**
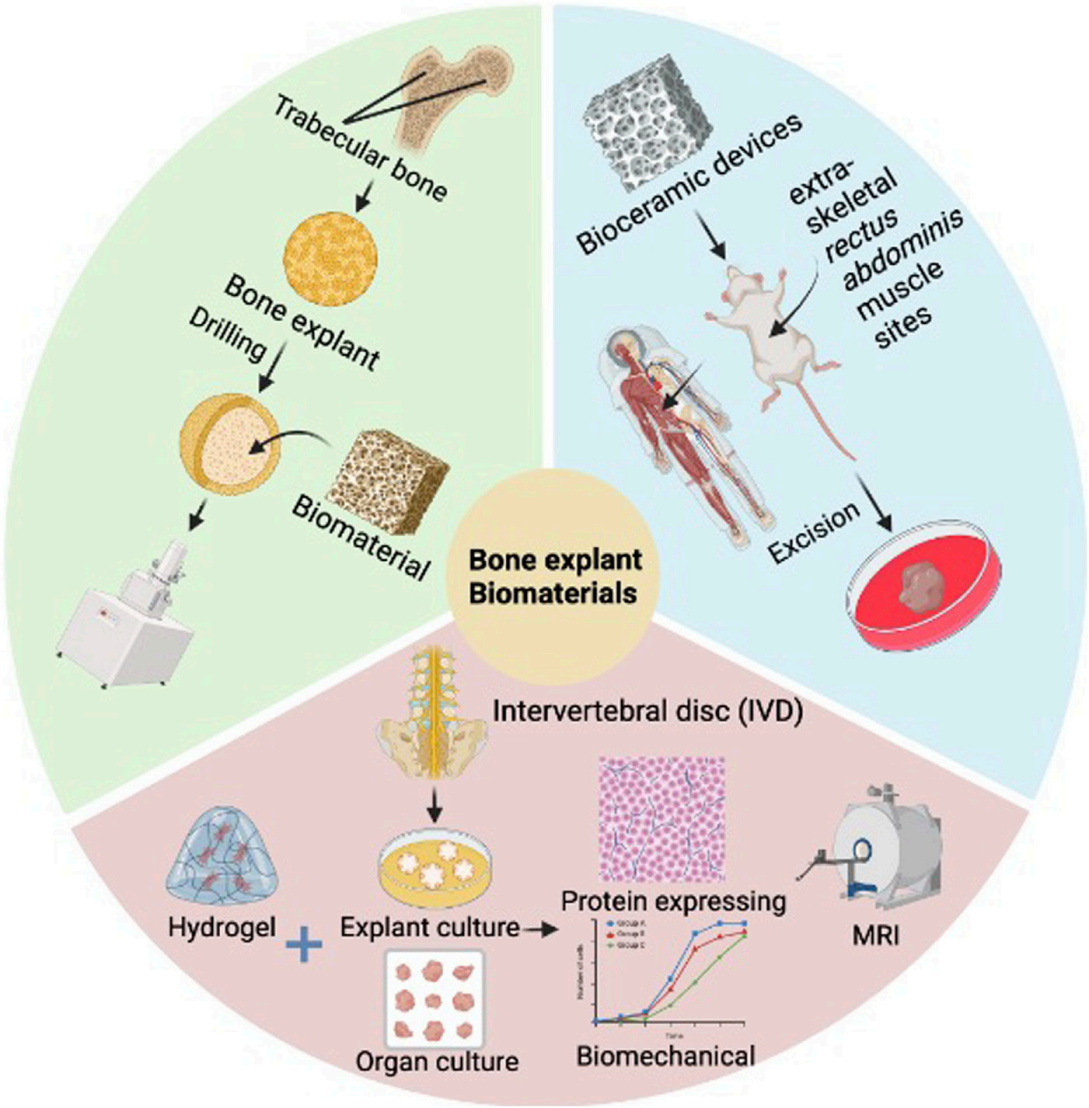
Application of tissue explant culture and biomaterials in bone diseases. This figure shows trabecular bone explants filled with biomaterials, osteogenic organoids utilizing muscle biomaterials, and hydrogel injection in an IVD tissue explant culture model. The combination of tissue explant culture and biomaterials provides an important experimental platform and technical means for studying the developmental mechanisms of bone organs, construction of disease models, drug screening and regenerative medicine treatment. Image created with BioRender (www.biorender.com).

The use of tissue explant cultures and biomaterials in bone organ research has been a crucial tool for a wide range of studies. In 2020, a new method has been proposed utilizing human trabecular bone explants filled with chitosan/gel/hydroxyapatite biomaterials to determine the osseointegration process *in vitro* and *in vivo* (Przekora et al., 2021). This study highlights the potential of tissue explant cultures to assess bone implant biocompatibility while avoiding the need for animal testing. Meanwhile, a proof-of-concept study of trauma-induced *in vitro* tissue survival was conducted using a muscle biomaterial-based osteogenic organoid system (He et al., 2020). To advance bone tissue engineering applications, this study examined tissue behavior and cell survival using a novel *in vitro* biomaterial organoid bioreactor system based on skeletal muscle tissue. In 2023, the regenerative potential of injectable hydrogels in inhibiting catabolic protein expression and promoting human nucleus pulposus cell matrix expression was studied in a tissue explant culture model (Cherif et al., 2023). This study focuses on the regeneration of naturally degenerated human intervertebral discs using a loaded organ culture model. Additionally, Osteoarthritis Models: From Animals to Tissue Engineering (2023) discusses the differentiation of bone organ tissue from stem or progenitor cells through appropriate induction, emphasizing the importance of biomaterial-based 3D microstructures in tissue engineering (Dou et al., 2023). In bioprinting for bone tissue engineering, the utilization of different types of biomaterials, cells, and growth factors has also been explored (Yazdanpanah et al., 2022). In summary, the combination of tissue explant culture and biomaterials holds great promise for advancing bone organ development and regenerative medicine research (Figure 2).

Table 2 compares the main advantages and disadvantages of stem cell models and tissue explant culture. Stem cell models have high differentiation potential and scalability and are suitable for disease modeling and drug screening. They can be genetically modified but may lack complex tissue microenvironments. Tissue explant culture maintains the original tissue structure and is closer to *in vivo* conditions but has a limited lifespan and is not easy to expand. These two comparisons are of great reference value for choosing the most suitable research method.

**TABLE 2:** The advantages and disadvantages between stem cell models and tissue explant culture.

	Stem cell models	Tissue explant culture
Definition	Cultures derived from stem cells, capable of differentiating into various cell types	Cultures established from fresh tissue samples, maintaining original tissue architecture
Advantages	1. High differentiation potential: hiPSCs and hESCs in particular are able to differentiate into any cell type 2. Scalability and renewability: Stem cells can be expanded indefinitely, providing a continuous source of cells 3. Genetic modification: Stem cells can be genetically edited to study specific diseases or functions 4. Disease modeling: Suitable for studying disease mechanisms, drug screening, and regenerative medicine 5. Heterogeneity: Large numbers of cells can be generated for high-throughput screening and large-scale studies	1. Preserve the original structure of the tissue: including cell-cell interactions and tissue-specific microenvironment 2. Reflect *in vivo* conditions: can more realistically reflect the physiological state and function of in vivo tissues 3. Short-term functional studies: suitable for observing acute reactions and functional analysis 4. Direct translation potential: tissues can be directly used for research without complex culture processes 5. Simple initial setup: no complex differentiation and maintenance conditions are required
Disadvantages	1. Lack of full tissue complexity: may not fully reproduce the in vivo tissue microenvironment 2. Complex differentiation protocols: the differentiation process of different cell types is complex and highly variable 3. Ethical issues: ethical issues may be involved, especially when using hESCs 4. Genetic and epigenetic abnormalities: prolonged culture may lead to genetic and epigenetic changes 5. High cost: the cost of culturing and maintaining stem cells is high	1. Limited lifespan: Tissue explants have a natural life cycle and are susceptible to degradation and loss of function 2. Non-renewable: Once used, it is not possible to re-expand or generate new samples 3. Limited genetic manipulation: Genetic modification is difficult and usually only pre-existing genetic modifications can be used 4. Individual variability: Individual differences between samples may lead to inconsistent results 5. Ethical issues: Ethical issues may be involved when obtaining human or animal tissues

## 4 Bone organoid types

Recent advances in the field of organoid research have led to the development of various types of bone organoids (Table 3). Cartilaginous organoids are primarily used to support and protect body structures such as the pinnae and nose. In 2020, researchers successfully used mouse induced pluripotent stem cells to create osteochondral organoids to simulate cartilage-bone interactions (Perez et al., 2018). Myeloid organoids are responsible for hematopoietic and immune functions. Olijnik et al., 2024. proposed a protocol to generate human myeloid organoids from induced pluripotent stem cells that combines multiple hematopoietic and stromal elements for disease modeling and drug discovery (Aude-Anais et al., 2024). Similarly, researchers from Radboudumc and Eindhoven University of Technology successfully intertwined various bone cells to create an organoid capable of independently generating new woven bone (Akiva et al., 2021; Radboudumc, 2021). In addition to woven bone organoids, trabecular bone organoids have also been developed. Through the guidance of osteoblasts to generate mineralized bone tissue and develop a phenotype resembling that of bone lining cells, trabecular bone organoids mimic trabecular bone plants, providing a model for studying bone structure and function (Chen et al., 2022a). The natural fracture healing process involves the formation of a cartilage intermediate (callus), which provides mechanical stability, enhances vascular regeneration, and recruits osteoprogenitor cells to induce bone formation. Researchers designed callus organoids using bone marrow stem cell-loaded hydrogel microspheres for rapid bone regeneration, achieving efficient bone regeneration within 4 weeks and highly outlining the involvement of callus in the endochondral ossification process of diverse cellular compositions and behaviors (Xie et al., 2022). Callus organoids are engineered to replicate the callus that forms during the natural healing process. Researchers demonstrate the development of engineered callus organoid bioassemblies that exhibit predictive long bone healing *in vivo* (Nilsson Hall et al., 2020).

**TABLE 3:** Summary of bone organoids.

Bone organoid type	Cell source	Year	Latest applications	References
Cartilaginous	BMSCs	2024	Basement membrane extract enhances the endochondral ossification phenotype of BMSCs-based cartilage organoids, indicating the potential for improving the functionality of cartilaginous tissues	Khan et al. (2023)
Woven bone organoids	BMSCs	2021	The differentiation of human BMSCs into a functional 3D self-organizing co-culture of osteoblasts and osteocytes, creating an organoid for early stage bone (woven bone) formation	Akiva et al. (2021)
Trabecular bone organoids	Primary female osteoblastic and osteoclastic cells	2021	The dense nature of trabecular bone allows for the detection of compositional gradients based on density, highlighting the potential for studying localized bone remodeling	Iordachescu et al. (2021)
Bone marrow organoids	hiPSCs	2024	Generating multilineage bone marrow organoids from hiPSCs, offering a method for disease modeling and drug discovery in the context of hematopoiesis.	Aude-Anais et al. (2024)
Callus organoids	BMSCs	2022	Engineering osteo-callus organoids for rapid bone regeneration using bone marrow-derived stem cell-loaded hydrogel microspheres and digital light-processing printing technology.	Xie et al. (2022)

Notes: BMSCs: Human bone marrow mesenchymal stem cells; 3D, Three-dimensional; hiPSCs, Human induced pluripotent stem cells.

## 5 Application of organoids in bone diseases

**FIGURE 3:**
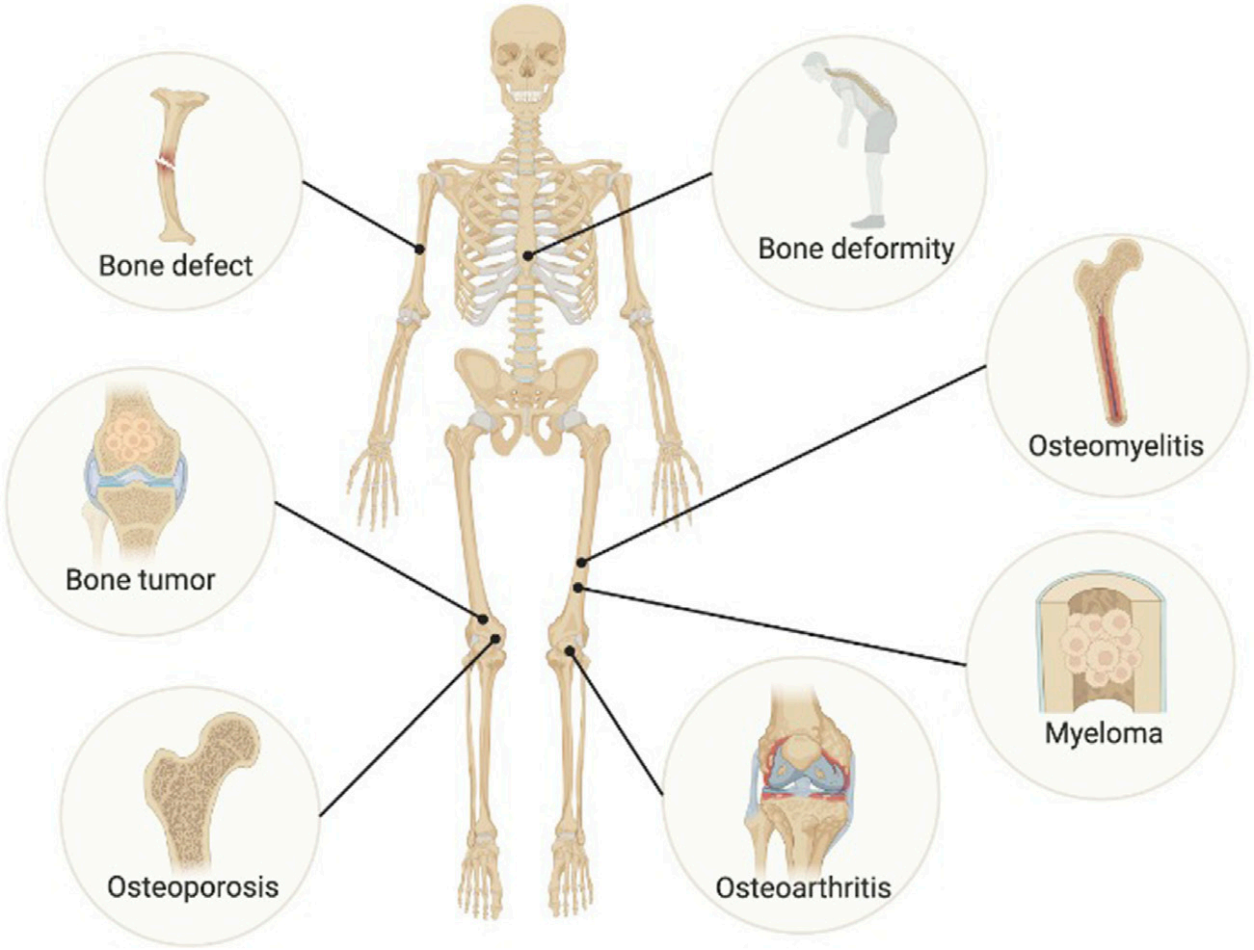
Disease modeling of bone organoids. Image created with BioRender (www.biorender.com).

Bone organoids more accurately replicate physiological and pathological conditions. To date, various ideas and methodologies have demonstrated tremendous potential for creating disease organoid models, and some bone disease organoids have been produced (Figure 3).

### 5.1 Osteoarthritis model

OA is a common degenerative joint disease characterized by pathological changes in both cartilage and bone. Connor et al. (2020) demonstrated the use of mouse induced pluripotent stem cells to create osteochondral organoids to model cartilage-bone interactions, an innovative approach that is promising for studying pathological changes in cartilage and bone associated with OA. Despite the paucity of studies on organoids in the skeletal system, especially cartilage tissue, the 2022 findings on the inhibition of FOX1 and FOXO3 in osteoarthritis progression highlights the potential of cartilaginous organoids as disease models (Yu et al., 2022). Furthermore, trabecular bone organoids have been proposed as models to study the regulation of bone resorption in diseases such as osteoarthritis (Park et al., 2021). Organoid models provide new possibilities for osteoarthritis research.

### 5.2 Osteoporosis model

Osteoporosis is a prevalent metabolic bone disease marked by bone loss, microstructural degradation, and increased fragility. *In vitro* osteoporosis models can be more easily and conveniently constructed by enhancing osteoclast activity and proportion in bone organoids, and by simulating reduced mechanical stimulation using microgravity bioreactors and similar techniques. *In vitro* culture of osteoporotic organoids has been considered a cost-effective and time-saving method for studying bone and joint diseases (Frenz-Wiessner et al., 2023). Additionally, trabecular bone organoids have been developed in 2021 to study the regulation of bone remodeling activities and address issues such as osteoporosis and reduced bone density (Iordachescu et al., 2021). The development of a 2022 “three-in-one” bone repair strategy for osteoporotic fractures involves the use of bone organoids to promote defect repair and address bone loss (Chen et al., 2022b).

### 5.3 Bone tumor model

Primary or secondary cancers of the human skeletal system and its associated tissues are referred to as bone tumors (Zhou et al., 2021). Research on bone tumors frequently depends on tumor cell lines and animal models. However, culturing tumor cell lines *in vitro* can lead to the loss of the tumor’s genetic heterogeneity, as they are removed from their original microenvironment. Establishing animal models requires an expensive and lengthy process. In contrast, tumor organoids better maintain the phenotypic and genetic features of the original tumor and can be expanded and cultured *in vitro*. In addition, tumor organoids can also demonstrate the occurrence and development of tumors. Recent research in 2023 shows that next-generation sequencing and organoid models have been used to study bone cancers, including osteosarcoma, using 3D culture models (Rausch et al., 2023).

### 5.4 Bone defect model

A bone defect is characterized by a partial or complete loss of bone tissue, typically resulting from trauma, infection, tumor excision, or other bone diseases. In 2019, Tan et al. developed a supramolecular hydrogel containing SDF-1 and BMP-2 that significantly promoted periodontal bone regeneration in rats (Tan et al., 2019). This approach resulted in high bone regeneration rates, demonstrating the potential of organoids in bone defect healing. In 2022, researchers successfully designed callus organoids that can rapidly regenerate bone in a short time, demonstrating the efficiency of this method in large bone defects (Xie et al., 2022). Furthermore, in 2024 Cardier et al. (2024) developed an osteogenic organoid to induce bone formation in patients with congenital tibial pseudarthrosis, demonstrating the potential of organoids to promote osteogenesis in challenging cases. These studies demonstrate the potential of organoids to enhance bone healing through various mechanisms, including growth factor release, cell differentiation, and tissue engineering strategies.

### 5.5 Bone marrow disease models (myeloma, osteomyelitis)

Osteomyelitis is a severe inflammation of the bones and bone marrow, often caused by infections such as *Staphylococcus aureus* (Hofstee et al., 2020). In the treatment of osteomyelitis, bone organoids can be used as a bioactive material to repair and regenerate damaged bone tissue. Bone organoids can also provide a suitable microenvironment that helps attract and direct the body’s own stem cells or other repair cells to promote the development and maintenance of bone tissue. Recent research has focused on developing advanced models to study the pathological mechanisms of osteomyelitis. One of the latest models is the 3D osteomyelitis model, which involves culturing bone or bone mimics with host cells to simulate the disease process (Hofstee et al., 2020).

Myeloma is a blood tumor that originates from plasma cells in the bone marrow and manifests as bone destruction, anemia, hypercalcemia, and kidney damage. Recent research has focused on developing 3D bone organoids to study myeloma bone disease (MBD) and potential treatment options. Visconti et al. (2021) aimed to create a well-characterized 3D bone organoid model for this purpose. Multiple myeloma is a hematologic malignancy primarily found in the bone marrow microenvironment. Human myeloid organoids have been used to model cancer-induced perturbations, demonstrating their utility in understanding disease (Azar et al., 2021). Patient-derived 3D models of multiple myeloma have also been developed for personalized medicine in 2022, using bone marrow organoids to culture patients’ malignant cells (Lourenço et al., 2022). Overall, the combination of advanced bone marrow disease models and bone organoids furthers our understanding of bone infection and may provide more effective treatment options for patients.

### 5.6 Bone deformity model

Bone deformity refers to the abnormal development of bones in form or structure, potentially resulting in irregular shapes or functional impairments. Bone organoids can be employed to repair bone defects and deformities caused by trauma, congenital abnormalities, or other factors. By implanting bone organoids into the affected area, the growth and repair of new bone tissue can be promoted, thereby improving the form and function of bone deformities. It can provide patients with personalized and effective treatment plans and provide new ways for research and understanding of diseases.

## 6 Drug screening

Most medications undergo extensive research, including both *in vivo* and *in vitro* testing, before gaining clinical approval. This process also applies to drugs used in the treatment of bone diseases, such as those with angiogenic, anti-inflammatory, antiresorptive, and bone-growth-promoting properties. However, in the traditional drug development methods, there are major shortcomings such as long organ toxicity assessment period and high cost (Horvath et al., 2016). The use of organoids for drug testing can, on the one hand, avoid errors caused by the huge biological differences between experimental animals and humans; on the other hand, organoids can enable high-throughput screening of drugs due to their availability and scalability. One of the challenges of utilizing organoids for drug screening is the need to create large numbers of homogeneous organoids suitable for high-throughput screening. Technologies like immersion bioprinting have been developed to overcome this limitation, allowing for the generation of tissue organoids in multiwell plates and thereby increasing the throughput of screening processes (Maloney et al., 2020). In addition, advances in patient-derived organoid culture platforms have optimized conditions for drug screening, allowing organoids to reach the optimal stage of drug testing in a short period of time (Nie et al., 2021). Self-assembled human skeletal organoids derived from bone and cartilage tissue have shown spontaneous polarization of their components, providing valuable models for disease modeling and drug testing (Abraham et al., 2021). In conclusion, bone organoids have important application potential in drug screening. By simulating the structure and function of bone tissue *in vivo*, bone organoids can give a more accurate *in vitro* research platform for evaluating the therapeutic efficacy and toxicity of drug candidates for bone-related diseases.

## 7 Regenerative medicine

Clinically, large-area bone defects that cannot heal on their own are typically addressed through autologous bone grafting, allogeneic bone grafting, or the use of artificial bone materials. However, autologous bone transplantation has limitations, including a restricted supply of material and the need for additional surgeries. Allogeneic bone transplantation carries risks such as disease transmission and immune rejection. Meanwhile, artificial bone materials like bioceramics and bone cement often lack osteoinductive properties and have issues with degradation rates. Problems such as mismatch with bone regeneration (Crispim and Ito, 2021). Bone organoids have become a potential solution for bone regeneration and repair, providing prospects for construction and application in various research fields such as organ development, drug screening, and mechanism research (Chen et al., 2022a). Research has demonstrated that callus organoids can be successfully engineered to promote quick and efficient bone repair, demonstrating the potential of bone organoids in tissue engineering regenerative medicine research (Xie et al., 2022). In addition, the application of organoids in skeletal disease research has shown promising results, highlighting the status and prospects of using organoids to understand and treat skeletal diseases (Wu et al., 2023a). Strategies targeting the microenvironment have also been proposed to guide advanced bone regeneration, emphasizing the importance of considering the surrounding environment in promoting effective bone repair (Hao et al., 2023). The aforementioned research indicates that bone organoids hold significant promise for use in OA *in vitro* modeling and regenerative medicine.

## 8 Latest technological advancements

**FIGURE 4:**
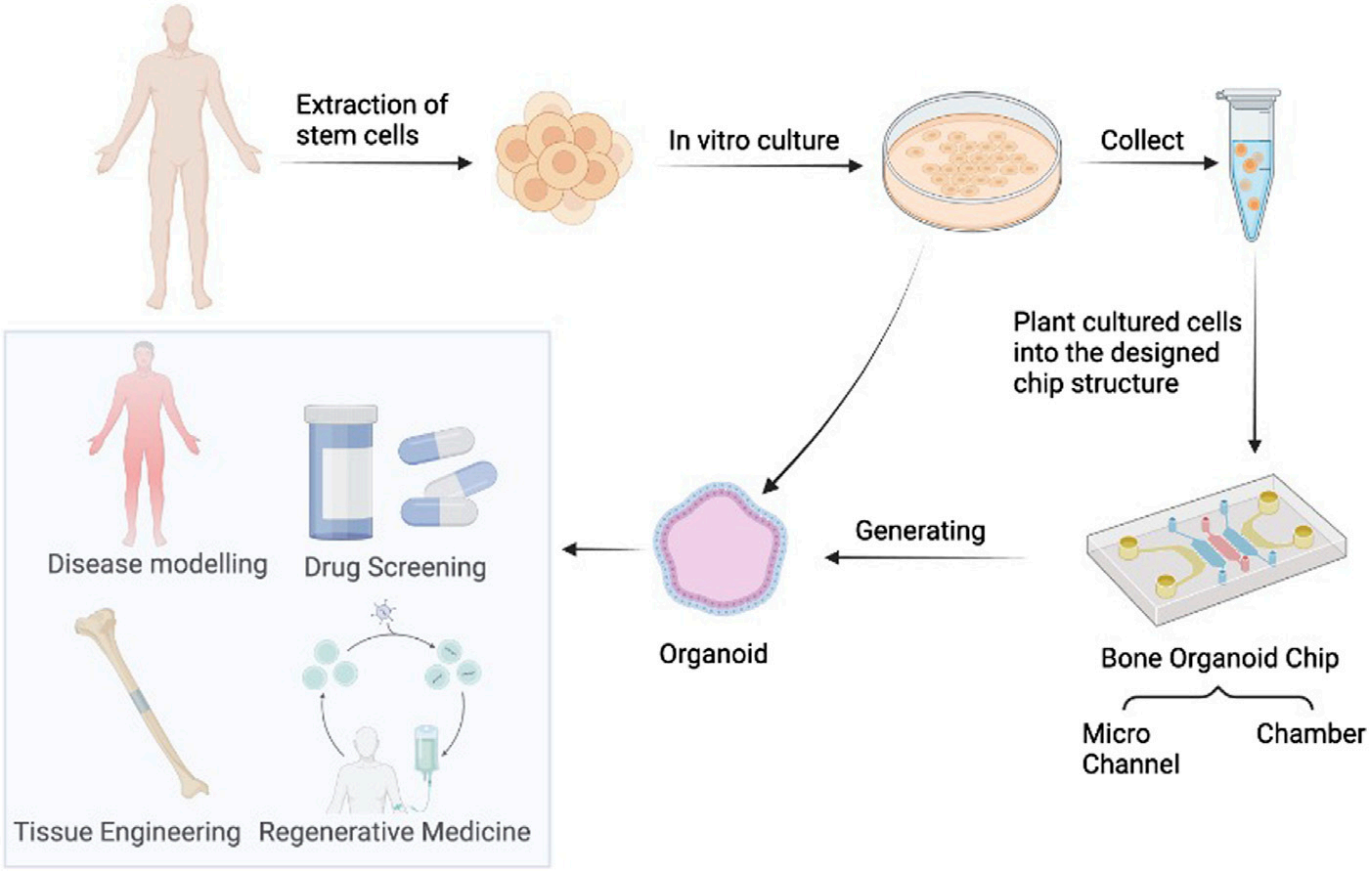
Bone Organoids and organoids-on-chips. These generated organoids have potential for drug screening, disease modeling, regenerative medicine, and tissue engineering. Image created with BioRender (www.biorender.com).

Recent technological advances in bone organoids encompass biomaterial-based 3D printing, advanced imaging technologies, and innovations in bone organoid chips. 3D printing technology enables precise control over the shape and structure of bone organoids, facilitating personalized customization and enhancing the efficiency and success rate of bone regeneration. In 2021, Researchers have explored the application of 3D printed bone tissue engineering scaffolds in the field of stem cells (Su et al., 2021). They discussed the advantages of 3D printing technology over traditional methods and identified some limitations and future research directions in the field. In the study of bone organoids, the use of advanced imaging techniques is critical to understanding their structure, function, and growth processes. In recent years, various advanced imaging technologies such as MRI (magnetic resonance imaging), CT (computed tomography), micro-CT (micro-computed tomography), PET (positron emission tomography), optical imaging technologies (such as fluorescence microscopy, co-focusing microscope) and so on are widely used in the study of bone organoids. Bone organoid chip is a miniaturized device used to simulate the microenvironment and function of human bone tissue. On a chip, bone organoids can introduce blood perfusion and joint synovial fluid stimulation through microfluidic technology, and accurately simulate the biomechanical properties of bone tissue. In addition, integrating different organoids on a chip can also establish musculoskeletal systems or even more complex systems for studying multi-system diseases or testing drug toxicity (Skardal et al., 2017). By using bone organoid chips, researchers can simulate the physiological and pathological states of bone tissue in the body, such as bone cell proliferation, differentiation, bone matrix deposition, and interaction with the immune system (Figure 4). This technology helps to gain a deeper understanding of bone biological processes, study the mechanisms of bone-related diseases, evaluate the efficacy and toxicity of drugs, and develop personalized treatments. Advances in these technologies will promote the application of bone organoids in bone disease treatment and regenerative medicine and provide more effective solutions for clinical treatment.

While these technological advancements have significantly improved the development and application of bone organoids, several critical areas require further exploration to move beyond the current state of the art:

Integration of Multimodal Approaches: The integration of 3D printing, advanced imaging, and organoid chip technology into a single, cohesive platform is still in its infancy. Future research should focus on combining these technologies to create more comprehensive and realistic models of bone tissue that can better simulate *in vivo* conditions.

Real-Time Monitoring and Dynamic Modelling: Current imaging techniques are often limited to static snapshots, which do not fully capture the dynamic processes occurring within bone organoids. Developing real-time monitoring systems and dynamic modeling approaches will be crucial for advancing our understanding of bone biology and disease mechanisms.

Scalability and Clinical Translation: While 3D printing and organoid chips offer promising avenues for personalized medicine, scaling these technologies for widespread clinical use remains a challenge. Addressing issues related to scalability, reproducibility, and cost-effectiveness will be essential for translating these innovations from the lab to the clinic.

Multiscale and Multifactorial Models: Existing organoid models often lack the complexity needed to study multi-system interactions, such as the interplay between bone tissue and immune responses. Developing multiscale and multifactorial models that can incorporate these interactions will enhance the relevance of bone organoids for studying systemic diseases and evaluating therapeutic interventions.

By addressing these challenges, future research can push the boundaries of current bone organoid technology, leading to more effective and clinically relevant solutions for bone regeneration and disease treatment.

## 9 Challenges and limitations

As a potential 3D *in vitro* culture model, bone organoids offer advantages that traditional 2D cell cultures and animal models lack. They hold significant promise in disease modeling, drug screening, regenerative medicine, and basic research. However, despite their potential, bone organoids still face some limitations. First, the cell types used in cultivating bone organoids are relatively simple, with most being limited to replicating specific bone functions, such as osteogenesis or bone marrow hematopoiesis. Bone tissue has a highly complex milieu that includes the circulatory system, extracellular matrix, and many different cell types. The complexity of this environment is challenging for existing organoid models to fully replicate, which has certain limits when it comes to replicating real bone tissue. To fully replicate the complexity of bone tissue, further research and advancements in cell co-culture techniques, 3D scaffolds materials, and bioreactor design are essential. Scaffold-free methods used to generate bone micro-organoids can be considered an extension of 3D printing technology, offering significant potential for the application of multifunctional bone tissue. Vascularization of bone organoids is a major challenge. Due to the lack of a vascular network, bone organoids cannot obtain sufficient nutrients through diffusion of blood and tissue fluid for cells beyond 200 μm from the capillaries (He et al., 2022). Due to the lack of a vascular network, the nutrient and waste exchange of organoids mainly relies on diffusion, which is inefficient in larger organoids and may lead to hypoxia and death of internal cells. Researchers are exploring a variety of technologies including microfluidic systems, genetically engineered vascularization, and bioprinting. In addition, co-culturing of endothelial cells or stimulating angiogenesis using VEGF, FGF, etc. may be expected to solve this problem.

Secondly, the construction of bone organoids lacks standardization. The process of producing organoids is complex and involves numerous variables, such as the source of cells, the components of the culture medium, and the materials used to create the 3D scaffold. The homogeneity and repeatability of organoids across laboratories are hampered by the difficulties in controlling these characteristics. Improving the repeatability and dependability of organoid models requires the establishment of consistent policies and norms. Organoids have demonstrated significant potential in basic research, but their translation into clinical therapies is still hindered by several challenges. This covers the immunological reaction and biocompatibility discrepancies between organoids and human tissues as well as methods for guaranteeing the uniformity and caliber of organoids produced on a wide scale. Innovative thinking and interdisciplinary cooperation are needed to solve these issues.

In addition to the difficulties faced in the formation of bone organoids, effective treatments need to be explored and identified to address bone-related diseases, especially bone-related cancers. Combined with CRISPR-Cas9 gene editing technology, disease-related genes can be screened, knocked out, or knocked in bone organoids, which can help to reproduce bone diseases, explore pathogenesis, and conduct gene-targeted therapy (Hendriks et al., 2020). Establishing a patient-derived bone tumor organoid biobank may also promote research on the effectiveness and safety of anti-tumor drugs (Jacob et al., 2020). As organoid technology continues to mature, bone organoids are expected to play a greater role in related fields.

Although bone organoids still encounter numerous challenges and limitations in their development and application, they exhibit significant potential in the treatment of bone diseases and regenerative medicine. With ongoing research and the advancement of new biomaterials like hydrogels, bone organoids are anticipated to surpass traditional matrix gels, enabling the more effective generation and maintenance of complex, multi-cellular bone organoids. Through continuous technological innovation and multidisciplinary cooperation, it is expected that these obstacles will be overcome in the future, and the research and application level of bone organoids will be further improved, providing more reliable and efficient tools for the diagnosis and treatment of bone diseases.

## 10 Conclusion and prospective

The field of bone organoids is rapidly evolving, with researchers exploring a variety of methods to generate these complex structures. The development and application of bone organoids hold great promise in advancing the field of bone regeneration and repair, offering innovative solutions for bone diseases and injuries. Further research in this area is critical, particularly to address existing limitations and to advance the clinical applications of bone organoids for enhancing bone repair and regeneration. It is essential to focus on improving the scalability of organoid technology and ensuring that the models accurately mimic human bone tissue. Additionally, exploring how these organoids can be effectively utilized for treating bone diseases is necessary.

In conclusion, bone organoids not only hold significant promise for advancing basic research but are also expected to bring revolutionary changes to future clinical applications. Our review highlights the latest advancements and identifies key areas for future research, emphasizing the transformative potential of bone organoids in addressing bone diseases and advancing the field of bone regeneration and repair. Continued innovation and interdisciplinary collaboration will be crucial in realizing the full potential of this technology.

## Abbreviations

2D, Two-dimensional; 3D, Three-dimensional; hiPSCs, Human induced pluripotent stem cells; hMSCs, Human mesenchymal stem cells; hESCs, Human embryonic stem cells; hPDCs, Human periosteum-derived cells; BMOs, Bone myeloid organoids; BMP4, Bone morphogenetic protein 4; IVD, Intervertebral disc; OA, Osteoarthritis; MBD, Myeloma bone disease; MRI, Magnetic resonance imaging; CT, Computed tomography; Micro-CT, micro-computed tomography; PET, Positron emission tomography.
